# Supplementary material for: Tooth Loss and Risk of Lung Cancer among Urban Chinese Adults: A Cohort Study with Meta-Analysis
Source: Cancers (Basel). 2022 May 14;14(10):2428. doi: 10.3390/cancers14102428 (PMC9140069; doi:10.3390/cancers14102428)
Supplement: Supplementary file 1 [file cancers-14-02428-s001.zip › cancers-1686359-supplementary.pdf]

Table S1: Characteristics of the study population by the number of tooth loss, SMHS and SWHS

	Men (SMHS)					Women (SWHS)				
	None (N=13,533)	1-5 (N=20,641)	6-10 (N=5,749)	>10 (N=9,945)	*p*-value†	None (N=12,953)	1-5 (N=22,739)	6-10 (N=6,919)	>10 (N=1,698)	*p*-value†
**Lung cancer cases**	87 (0.6)	211 (1.0)	86 (1.5)	229 (2.3)	<0.001	94 (0.7)	174 (0.8)	75 (1.1)	17 (1.0)	0.03
**Age,** [a] mean (SD)	55.5 (7.2)	59.7 (8.7)	64.5 (9.2)	68.6 (8.9)	<0.001	57.5 (6.5)	60.9 (7.9)	65.4 (8.5)	67.9 (8.3)	<0.001
**Education**										
Less than high school	4,425 (32.7)	8,078 (39.1)	2,536 (44.1)	5,015 (50.4)	<0.001	6,762 (52.2)	12,950 (57.0)	4,419 (63.9)	1,156 (68.1)	<0.001
Completed high school	5,890 (43.5)	7,731 (37.5)	1,776 (30.9)	2,704 (27.2)		4,420 (34.1)	6,808 (29.9)	1,629 (23.5)	354 (20.8)	
More than high school	3,218 (23.8)	4,832 (23.4)	1,437 (35.0)	2,226 (22.4)		1,771 (13.7)	2,981 (13.1)	871 (12.6)	188 (11.1)	
**Income**[d]										
Low	1,921 (14.2)	2,710 (13.1)	653 (11.4)	990 (9.9)	<0.001	1,683 (13.0)	3,226 (14.2)	1,129 (16.3)	337 (19.9)	<0.001
Medium	10,073 (74.4)	15,829 (76.7)	4,568 (79.5)	8,392 (64.4)		9,839 (76.0)	17,261 (75.9)	5,287 (76.4)	1,255 (73.9)	
High	1,539 (11.4)	2,102 (10.2)	528 (9.2)	563 (5.7)		1,431 (11.0)	2,252 (9.9)	503 (7.3)	106 (6.2)	
**Smoking Status**										
Never	4,003 (29.6)	6,303 (30.5)	1,797 (31.3)	3,073 (30.9)	<0.001	12,761 (98.5)	22,291 (98.0)	6,731 (97.3)	1,623 (95.6)	<0.001
Former	955 (7.1)	1,972 (9.6)	709 (12.3)	1,659 (16.7)		19 (0.2)	65 (0.3)	25 (0.4)	12 (0.7)	
Current	8,575 (63.4)	12,366 (59.9)	3,243 (56.4)	5,213 (52.4)		173 (1.3)	383 (1.7)	163 (2.4)	63 (3.7)	
**Pack-years,**[b] mean (SD)	20.6 (13.0)	23.2 (14.7)	26.9 (16.9)	30.0 (18.9)	<0.001	6.0 (8.1)	7.8 (10.6)	10.5 (10.8)	10.7 (10.9)	<0.001
**Alcohol consumption**[c]										
None	9,118 (67.4)	13,782 (66.8)	3,763 (65.5)	6,612 (66.5)	0.002	12,730 (98.3)	22,271 (97.9)	6,784 (98.1)	1,666 (98.1)	0.20
Low-to-moderate	2,691 (19.9)	4,206 (20.4)	1,179 (20.5)	1,922 (19.3)		169 (1.3)	374 (1.7)	111 (1.6)	28 (1.7)	
Heavy	1,724 (12.7)	2,653 (12.8)	807 (14.0)	1,411 (14.2)		54 (0.4)	94 (0.4)	24 (0.3)	4 (0.2)	
**BMI**										
Under weight, <18.5	516 (3.8)	720 (3.5)	242 (4.2)	554 (5.6)	<0.001	431 (3.3)	677 (3.0)	210 (3.0)	58 (3.4)	<0.001
Normal, 18.5-24.9	8,654 (63.9)	12,908 (62.5)	3,570 (62.1)	6,161 (61.9)		8,813 (68.1)	14,234 (62.6)	3,880 (56.1)	887 (52.2)	
Overweight, 25.0-29.9	4,053 (30.0)	6,459 (31.3)	1,803 (31.4)	2,956 (29.7)		3,279 (35.3)	6,768 (29.8)	2,377 (34.4)	635 (37.4)	
Obese, ≥30	310 (2.3)	554 (2.7)	134 (2.3)	274 (2.8)		430 (3.3)	1,060 (4.6)	452 (6.5)	118 (7.0)	
**COPD**[e]										
No	12,627 (93.3)	18,723 (90.7)	5,003 (87.0)	8,238 (82.8)	<0.001	11,529 (89.0)	19,887 (87.5)	5,903 (85.3)	1,432 (84.3)	<0.001
Yes	906 (6.7)	1,918 (9.3)	746 (13.0)	1,707 (17.2)		1,424 (11.0)	2,852 (12.5)	1,016 (14.7)	266 (15.7)	
**Menopausal status**										
Pre	-	-	-	-	N.A.	9,609 (74.2)	13,246 (58.3)	2,493 (36.0)	446 (26.3)	<0.001
Post	-	-	-	-		3,344 (25.8)	9,493 (41.7)	4,426 (64.0)	1,252 (73.7)	

LC, lung cancer; N, number; SD, standard deviation; BMI, body mass index; COPD, chronic obstructive pulmonary disease; N.A., not applicable.

†Differences between lung cancer cases and non-cases across baseline characteristics were evaluated by generalized linear models for continuous variables or chi-square test for categorical variables.

[a]Age at oral health assessment.

[b]Among current and former smokers.
[c]Number of total alcoholic drinks (1 drink=14 grams of ethanol) consumed per day were defined as none, low-to-moderate (>0 to ≤2 and >0 to ≤1 drink/day for men and women, respectively), and heavy (>2 and >1 drink/day, respectively).
[d]Annual personal income, low, medium, and high, defined as <4,000, ≥4,000 to <8,000, ≥8,000 yuan in the SWHS and <6,000, ≥6,000 to <10,000, ≥10,000 yuan in the SMHS, respectively.
[e]Ever diagnosed with emphysema or pulmonary tuberculosis or chronic bronchitis or asthma.

Table S2: Association of smoking status with risk of tooth loss, SMHS and SWHS

				Stratified Analysis by Number of Tooth Loss					
**Smoking status**	**None**	**Any**	**OR (95% CI)** [a]	**1-5**	**OR (95% CI)** [a]	**6-10**	**OR (95% CI)** [a]	**>10**	**OR (95% CI)** [a]
**Men**									
Never	4,003	11,173	1 (ref.)	6,303	1 (ref.)	1,797	1 (ref.)	3,073	1 (ref.)
Former	955	4,340	1.07 (0.98-1.18)	1,972	1.09 (0.98-1.20)	709	1.00 (0.86-1.16)	1,659	1.05 (0.91-1.21)
Current	8,575	20,822	1.25 (1.17-1.34)	12,366	1.20 (1.12-1.29)	3,243	1.44 (1.28-1.62)	5,213	1.54 (1.37-1.72)
**Women**									
Never	12,761	30,645	1 (ref.)	22,291	1 (ref.)	6,731	1 (ref.)	1,623	1 (ref.)
Former	19	102	1.18 (0.70-1.20)	65	1.20 (0.70-2.05)	25	0.83 (0.41-1.70)	12	1.63 (0.67-3.94)
Current	173	609	1.19 (0.96-1.48)	383	1.12 (0.90-1.40)	163	1.19 (0.86-1.66)	63	1.73 (1.08-2.78)

OR, odds ratio; 95% CI, 95% confidence interval; Ref., reference.

[a] Estimated using logistic regression models adjusted for age at tooth loss assessment, pack-years, education, income, alcohol consumption, BMI, history of COPD, and menopausal status (only for women).

Table S3: Association between tooth loss and lung cancer risk: sensitivity analysis excluding the first two years of followed-up after oral health assessment, SMHS and SWHS

Number of tooth loss	Incident cases, *n*	Person-years	Incidence rate[a]	Model 1[b] HR (95% CI)	Model 2[c] HR (95% CI)	Model 3[d] HR (95% CI)
**Men**						
None	66	90,932	72.6	1 (ref.)	1 (ref.)	1 (ref.)
1-5	160	136,748	117.0	1.32 (0.98-1.76)	1.22 (0.91-1.63)	1.22 (0.91-1.64)
6-10	68	37,079	183.4	1.76 (1.24-2.51)	1.45 (1.01-2.06)	1.44 (1.01-2.06)
> 10	159	62,109	256.0	2.19 (1.60-3.01)	1.63 (1.18-2.24)	1.61 (1.16-2.23)
p for trend				<0.001	0.002	0.002
**Women**						
None	78	102,492	76.1	1 (ref.)	1 (ref.)	1 (ref.)
1-5	138	178,481	77.3	0.85 (0.64-1.13)	0.85 (0.64-1.13)	0.86 (0.65-1.14)
6-10	58	53,539	108.3	1.01 (0.70-1.44)	1.00 (0.70-1.43)	1.02 (0.71-1.46)
> 10	13	13,106	99.2	0.87 (0.48-1.60)	0.85 (0.46-.155)	0.88 (0.48-1.62)
p for trend				0.86	0.79	0.90

HR, odds ratio; 95% CI, 95% confidence interval; Ref., reference.

[a]Incidence rate per 100,000 person-years.

[b]Adjusted for age at tooth loss assessment. Age at tooth loss assessment (entry) and age at censoring (exit) were treated as the time scale.

[c]Adjusted for age at tooth loss assessment, smoking status, and pack-years. Age at tooth loss assessment (entry) and age at censoring (exit) were treated as the time scale.

[d]Adjusted for age at tooth loss assessment, smoking status, pack-years, education, income, alcohol consumption, BMI, history of COPD, and menopausal status (only for women). Age at tooth loss assessment (entry) and age at censoring (exit) were treated as the time scale.

Table S4: Joint effect of tooth loss and smoking in association with lung cancer risk, SMHS and SWHS

	Incident cases, *n*	Person-years	Incidence rate[a]	Model 1[b]	Model 2[c]	Model 3[d]
				HR (95% CI)	HR (95% CI)	HR (95% CI)
**Men**						
Non-current smokers[e] & No tooth loss	25	33,535	74.5	1 (ref.)	1 (ref.)	1 (ref.)
Non-current smokers[e] & Tooth loss	157	101,102	155.3	1.16 (0.76-1.78)	1.09 (0.71-1.68)	1.08 (0.70-1.65)
Current smokers & No tooth loss	62	57,533	107.8	2.33 (1.45-3.72)	1.74 (1.06-2.87)	1.68 (1.02-2.76)
Current smokers & Tooth loss	369	135,780	271.8	3.45 (2.30-5.17)	2.46 (1.58-3.82)	2.33 (1.50-3.63)
p for trend				<0.001	0.03	0.04
**Women**						
Non-current smokers[e] & No tooth loss	92	101,268	90.8	1 (ref.)	1 (ref.)	1 (ref.)
Non-current smokers[e] & Tooth loss	250	240,869	103.8	0.87 (0.68-1.11)	0.87 (0.68-1.12)	0.88 (0.69-1.13)
Current smokers & No tooth loss	2	1,320	151.5	1.51 (0.37-6.11)	1.15 (0.28-4.74)	1.32 (0.32-5.46)
Current smokers & Tooth loss	16	4,623	346.1	2.57 (1.50-4.42)	1.71 (0.88-3.31)	1.88 (0.97-3.66)
p for trend				0.12	0.33	0.38

HR, hazard ratio; 95% CI, 95% confidence interval; Ref., reference.

[a]Incidence rate per 100,000 person-years.

[b]Adjusted for age at tooth loss assessment. Age at tooth loss assessment (entry) and age at censoring (exit) were treated as the time scale.

[c]Adjusted for age at tooth loss assessment, smoking status, and pack-years. Age at tooth loss assessment (entry) and age at censoring (exit) were treated as the time scale.

[d]Adjusted for age at tooth loss assessment, smoking status, pack-years, education, income, alcohol consumption, BMI, history of COPD, and menopausal status (only for women). Age at tooth loss assessment (entry) and age at censoring (exit) were treated as the time scale.

[e]Non-current smokers included never and former smokers.

Table S5: Characteristics of prospective studies included in the meta-analysis

No	Author	Source of Participants	Country	Study Enrollment	Follow-up Years	LC cases / Non-cases	Covariates Adjusted	Hazard Ratios / Odds Ratios (95% CIs)
1	Michaud DS[a]	Health Professionals Follow-Up Study (HPFS)	USA	1986-1986	Median: 17.7	678 / 47,697	age, ethnic origin, physical activity, history of diabetes, alcohol, BMI, geographical location, height, calcium intake, total calorific intake, red-meat intake, fruit and vegetable intake, vitamin D score, smoking history, and pack-years	*Number of teeth*<br>25–32: 1.00 (Ref.)<br>17–24: 1.34 (1.10–1.63)<br>0–16: 1.70 (1.37–2.11)
2	Hiraki A[a]	Aichi Cancer Center Study (ACCS)	Japan	2001-2005	N/A	909 / 1,818	age, sex, smoking and drinking status, vegetable and fruit intake, BMI, and regular exercise	*Number of teeth*<br>21: 1.00 (Ref.)<br>9-20: 1.02 (0.83-1.24)<br>1-8: 1.22 (0.94-1.57)<br>0: 1.54 (1.05-2.27)
3	Mai X	Women's Health Initiative Observational Study (WHI-OS)	USA	1993-1998	Mean: 6.8	754 / 76,731	age, smoking status, pack-years	*Edentulism*<br>No: 1.00 (Ref.)<br>Yes: 1.64 (1.07-2.52)
4	Michaud DS[a]	Health Professionals Follow-Up Study (HPFS)	USA	1986-1986	Until 2012	109 / 19,824	age, race, alcohol use, physical activity, history of diabetes, BMI, geographical location, height, NSAID use.	*Number of teeth*<br>25–32: 1.00 (Ref.)<br>17-24: 1.36 (0.76–2.42)<br>0-16: 1.47 (0.59–3.68)
5	Michaud DS[a]	Atherosclerosis Risk in Communities Study (ARIC)	USA	1996-1998	Mean: 14.7	226[b] / 7,240	age, field center, education level, smoking status, smoking duration, drinking status, BMI, and diabetes status, joint terms for sex	*Edentulism* [c]<br>No: 1.00 (Ref.)<br>Yes: 2.60 (1.65-4.08)

							and HRT use	
6	Yoon HS[a*]	Southern Community Cohort Study (SCCS)	USA	2002-2009	Until 2015	403 / 1,612	age, BMI, education, household income, COPD, alcohol drinking, smoking status, and pack-years	*Number of tooth loss*<br>None: 1.00 (Ref.)<br>1-4: 1.20 (0.70-2.03)<br>5-10: 1.22 (0.71-2.09)<br>> 10: 1.64 (1.00-2.69)

Abbreviations: BMI, body mass index; COPD, chronic obstructive pulmonary disease; HRT, hormone replacement therapy; LC, lung cancer; NSAID, Non-steroidal anti-inflammatory drugs; Ref., reference.

[a] Presented results by smoking status; current or former or never

[a*] Conducted additional analysis by smoking status in the previous study

[b] Included were incident cases of lung and bronchus cancer

[c] Presented the ARIC classification results

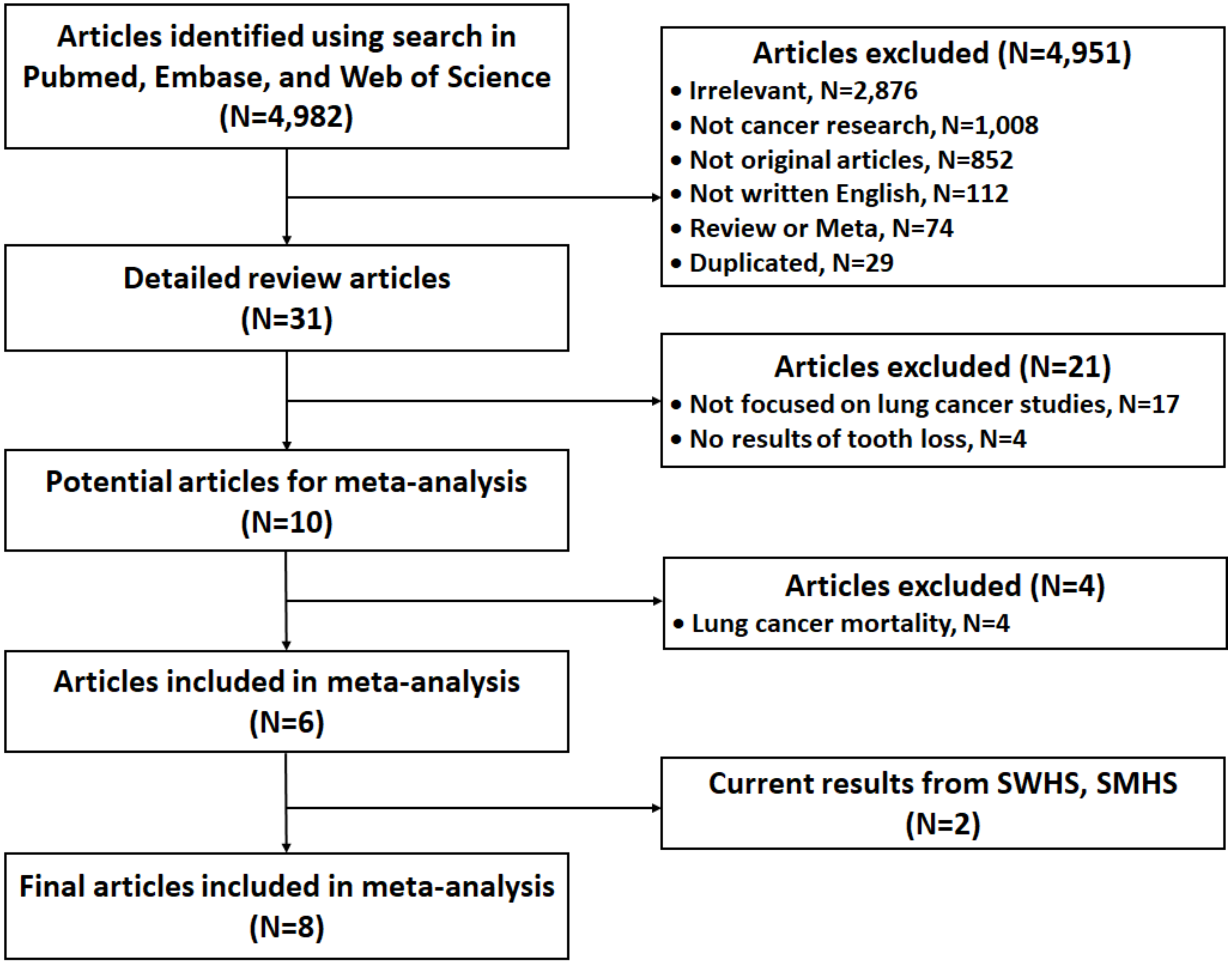

Figure S1. Article selection process for the systematic review and meta-analysis

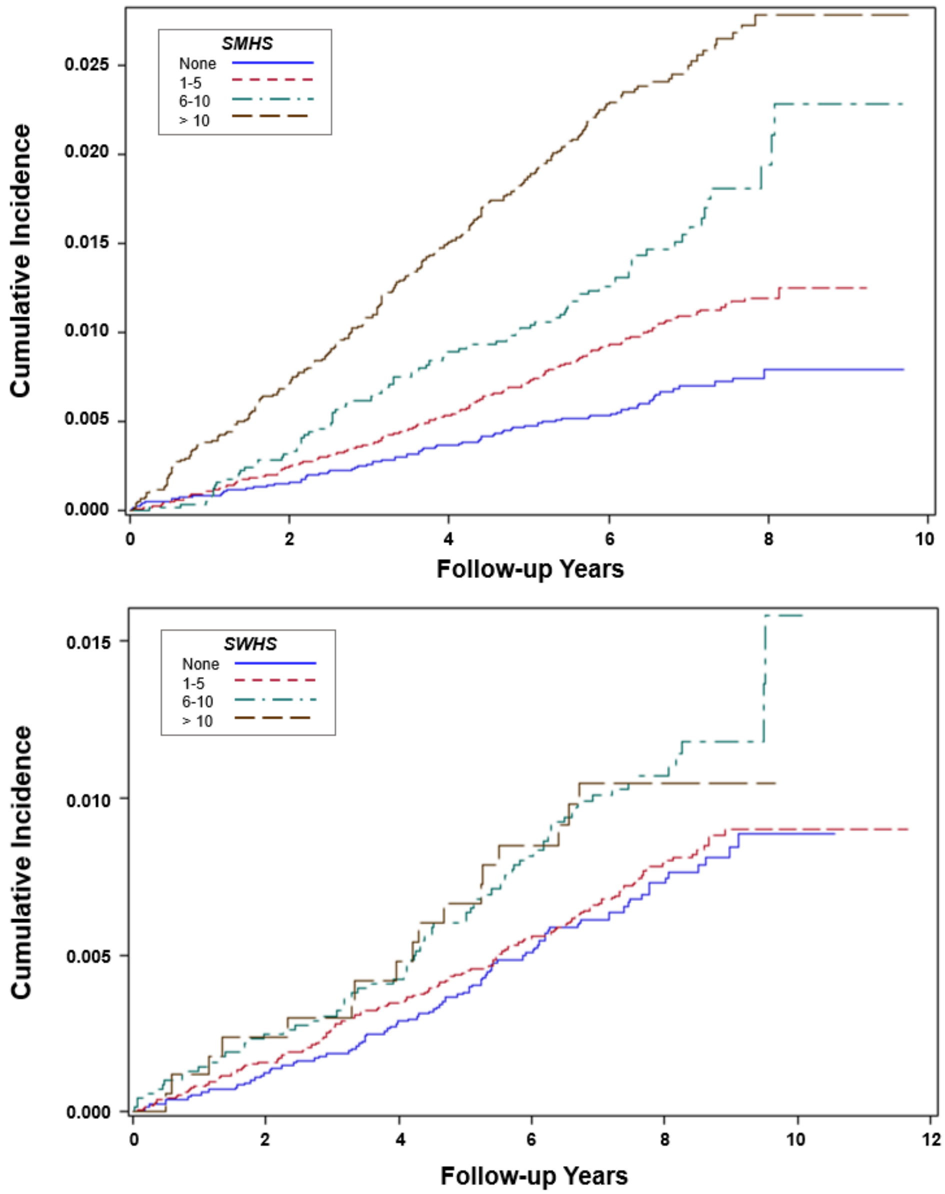

Figure S2. Cumulative incidence curves by number of tooth loss in Shanghai Men's and Women's Health Study

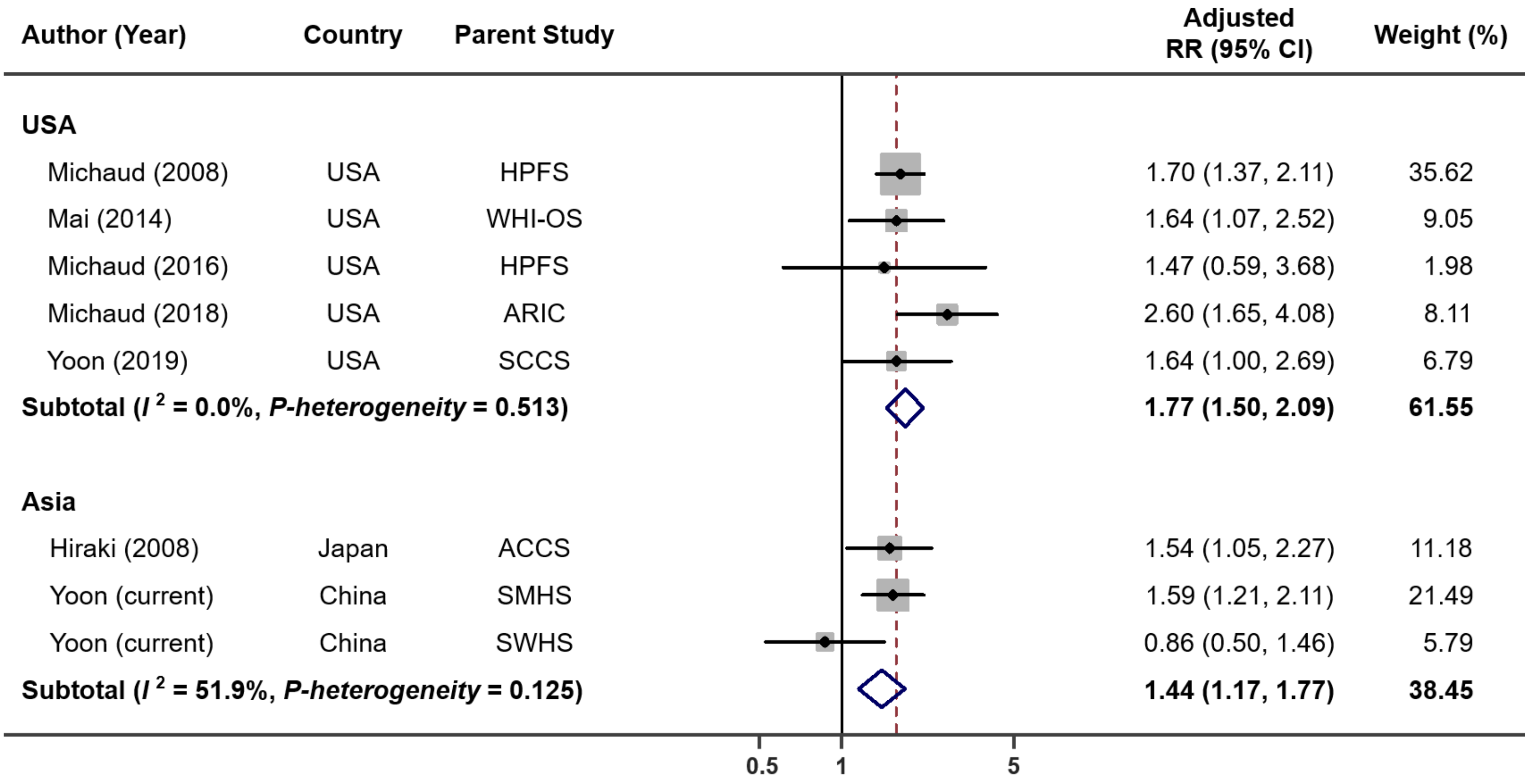

Figure S3. Lung cancer risk associated with tooth loss: Subgroup Analysis by studies in the US and Asia

Abbreviations: ACCS, Aichi Cancer Center Study; ARIC, Atherosclerosis Risk in Communities Study; HPFS, Health Professionals Follow-Up Study; RR, relative risk; SCCS, Southern Community Cohort Study; SMHS, Shanghai Men's Health Study; SWHS, Shanghai Women's Health Study; WHI-OS, Women's Health Initiative Observational Study.
